# The Wnt/β-catenin signaling pathway has a healing ability for periapical periodontitis

**DOI:** 10.1038/s41598-021-99231-x

**Published:** 2021-10-04

**Authors:** Haruna Naruse, Shousaku Itoh, Yuki Itoh, Takumi Kagioka, Makoto Abe, Mikako Hayashi

**Affiliations:** 1 Department of Restorative Dentistry and Endodontology, Osaka University Graduate School of Dentistry, 1-8 Yamadaoka Suita, Osaka, 565-0871 Japan; 2 Department of Oral Anatomy and Developmental Biology, Osaka University Graduate School of Dentistry, Osaka, Japan

**Keywords:** Biomaterials, Experimental models of disease, Dental diseases, Oral diseases

## Abstract

Various disease-related genes have recently been identified using single nucleotide polymorphisms (SNPs). This study identified disease-related genes by analyzing SNP using genomic DNA isolated from Japanese patients with periapical periodontitis. Results showed that the SNP in *LRP5* demonstrated a significant genotypic association with periapical lesions (Fisher’s exact test, *P* < 0.05). We constructed an in vivo murine periapical periodontitis model to confirm the Wnt/β-catenin signaling pathway’s role in developing and healing periapical periodontitis. We observed that administration of the Wnt/β-catenin signaling pathway inhibitor enlarged the periapical lesion. Moreover, applying lithium chloride (LiCl) to root canals accelerated periapical periodontitis healing. Histological analysis demonstrated that the expression levels of *Col1a1* and *Runx2* increased in the LiCl application group compared to that in the control group. Furthermore, many CD45R-positive cells appeared in the periapical lesions in the LiCl application group. These results indicated that LiCl promoted the healing of periapical periodontitis by inducing bone formation and immune responses. Our findings suggest that the Wnt/β-catenin signaling pathway regulates the development of periapical periodontitis. We propose a bioactive next-generation root canal treatment agent for this dental lesion.

## Introduction

Periapical periodontitis is an inflammatory disease of periapical tissues caused by an infection in the root canal system due to inadequate root canal treatment. The invasion of microbes from the root canal system into the periapical tissues induces host immune responses^1,2^. During the host defense reaction, the host’s cells produce various inflammatory and anti-inflammatory molecules, and some inflammatory molecules induce osteoclast differentiation^3^. Then, bone homeostasis is disrupted in case of periapical periodontitis, which promotes increased rates of bone resorption, resulting in the formation of periapical lesions that are observed as radiolucent areas^3^. Several disease-related genes have been identified for various multifactorial diseases using single nucleotide polymorphisms (SNPs) in recent years. In case–control studies, some researchers reported that *IL-1β* polymorphism is associated with periapical periodontitis^4–6^. Meanwhile, although several previous SNP studies reported inflammatory cytokines related to periapical periodontitis, only a few reports described other factors^7,8^. In the field of bone metabolism, studies reported that the SNP located in exon 18 of the low-density lipoprotein receptor-related protein 5 (LRP5, Wnt coreceptor) gene, caused due to an amino acid change (3989C>T, A1330V), is associated with osteoporosis among patients in Japan, Europe, America, and South America^9–18^. Therefore, in this study, we focused on the SNP of LRP5 related to bone metabolism.

The Wnt/β-catenin signaling pathway plays a significant role in the maintenance of bone homeostasis^19,20^. Wnt proteins bind to their receptor Frizzled and the coreceptor LRP 5/6, suppressing the phosphorylation of β-catenin using glycogen synthase kinase-3β (GSK-3β). The stabilized β-catenin accumulates in the cytoplasm. Accumulation and nuclear translocation of β-catenin enable the association with the transcription factor LEF-1/TCF and activate various target genes (canonical pathway). It has been reported that Li^+^ can activate the Wnt/β-catenin signaling pathway by inhibiting GSK-3β activity^21^. Experimental studies also reported that lithium chloride (LiCl) induces hard tissue formation in vivo and in vitro^21,22^. Conversely, there are also the report describing that the Wnt/β-catenin signaling pathway regulates the differentiation of T cells and B cells^23^. For example, in one study, the inhibition of the Wnt canonical pathway blocked the transition from double-negative T cells to double-positive T cells^24^. In another study, LEF-1-deficient mice exhibited defects in pro-B-cell proliferation and survival both in vitro and in vitro^23^. Periapical periodontitis is caused by the host’s defense against microbial invasion and alveolar bone resorption by disrupting bone homeostasis. Hence, we focused on the role of the Wnt/β-catenin signaling pathway in both immunity and bone homeostasis.

We hypothesized that the A1330V variant of LRP5 might be associated with apical periodontitis. In the present study, we selected individuals with periapical lesions measuring > 3 mm in diameter as the case group. We performed a genetic polymorphism analysis for these individuals and a control group (individuals with no apical lesion). We found that the A1330V variant of LRP5 was associated with periapical periodontitis. To confirm the Wnt/β-catenin pathway’s role in periapical periodontitis development, we practiced an in vivo murine periapical periodontitis model. Although the administration of the Wnt/β-catenin pathway inhibitor enlarged the apical lesion, applying LiCl into the root canals accelerated periapical periodontitis healing. Our findings demonstrated that the Wnt/β-catenin signaling pathway regulates periapical periodontitis development. We propose a novel therapeutic strategy for this dental lesion.

## Results

### LRP5 SNP is related to periapical periodontitis

The mean age of patients was 48.2 ± 20.3 years in the case group and 59.8 ± 17.4 years in the control group. The case group consisted of 19 males and 31 females, and the control group consisted of 12 males and 18 females. The mean diameter of the periapical lesion was 6.0 ± 2.7 mm. Table 1 shows the results of genotyping of *LRP5* SNP (A1330V) for the case and control groups. The frequency of C/T was statistically significant between the groups (case group vs. control group, *P* = 0.00396).

**Table 1:** Results of case–control comparisons for the association of SNPs in LRP5.

*LRP5* (rs3736228)	Genotype frequency		
	Case	Control	P value
C/C	0.36	0.54	NS
C/T	0.62	0.46	0.00396*
T/T	0.02	0.00	NS

A statistically significant difference occurred in C/T frequency (case group vs. control group, *P* < 0.05 as assessed by Fisher’s exact test).

### Inhibition of the Wnt/β-catenin signaling pathway spreads the periapical lesion

**Figure 1:**
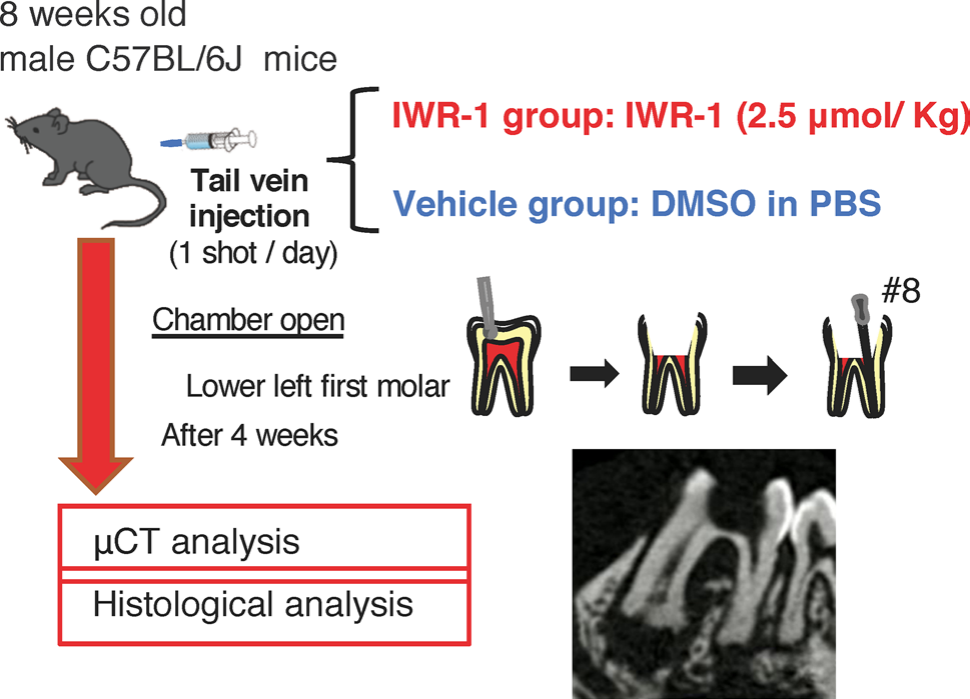
In vivo inhibition model of the Wnt/β-catenin signaling pathway. The pulp chamber of the lower first molar was exposed to the oral cavity. IWR-1 or DMSO in PBS was administered into the tail vein once a day from the day of pulp exposure. At 4 weeks after the first administration, the periapical lesions were evaluated by micro-CT and histological analysis. A representative image for the micro-CT is shown.

**Figure 2:**
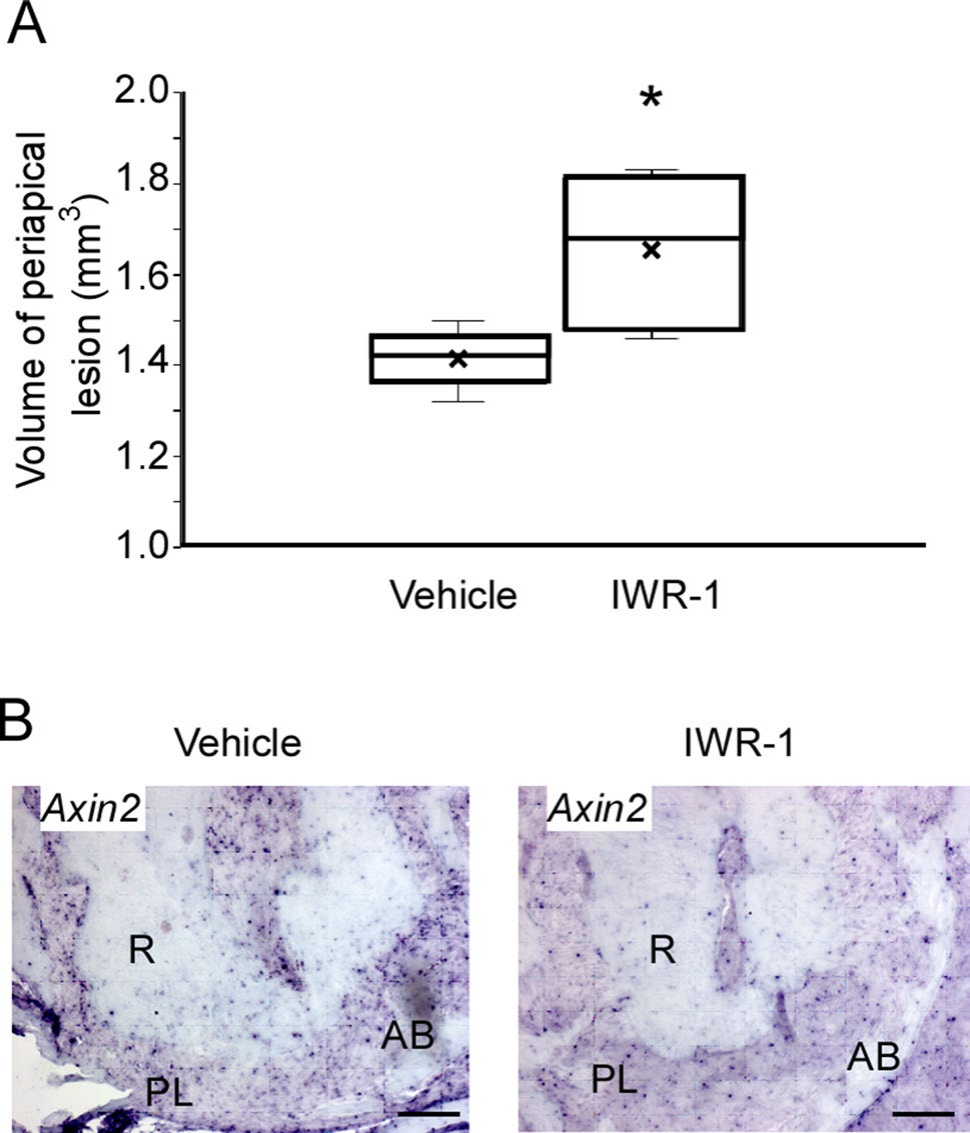
IWR-1 injection inhibited the Wnt/β-catenin signaling pathway and spread the periapical lesion. (**A**) The quantification of periapical lesion volumes of the vehicle group (white bar, mean ± SD, n = 4) and the IWR-1 treatment group (black bar, mean ± SD, n = 4). Two-tailed paired Student’s *t* test, *P < 0.05. (**B**) In situ hybridization for *Axin2* in the periapical lesion at 4 weeks after chamber opening. *R* root, *PL* periapical lesion, *AB* alveolar bone. Scale bars = 100 μm.

**Figure 3:**
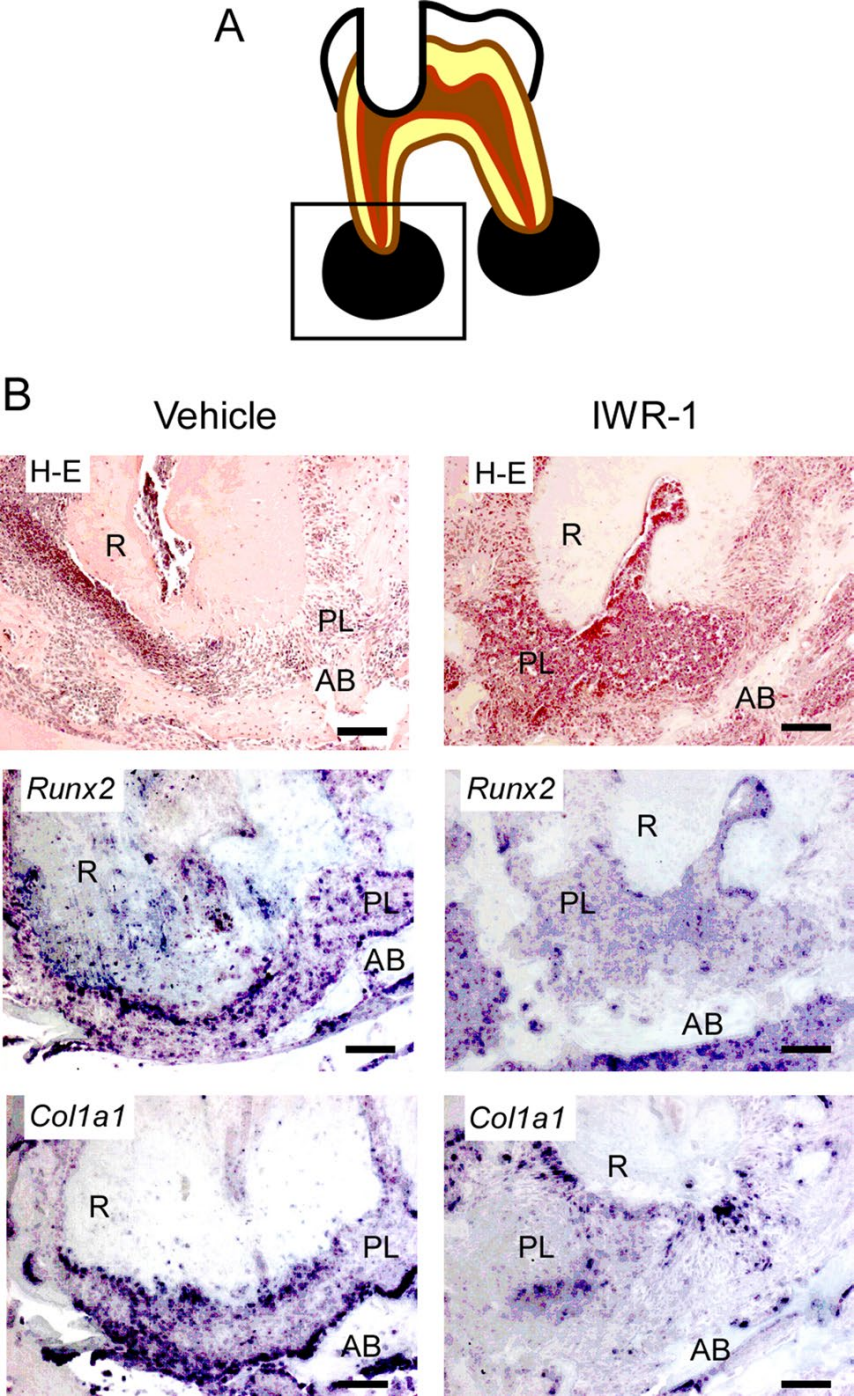
Inhibition of the Wnt/β-catenin signaling pathway resulted in suppressed expression levels of osteoblast differentiation markers. Periapical lesion tissues within a rectangular frame (**A**) of the vehicle group and the IWR-1 group were subjected to H–E staining and in situ hybridization for *Runx2* and *Col1a1* (**B**). *R* root, *PL* periapical lesion, *AB* alveolar bone. Scale bars = 100 μm.

The lower left first molar pulp was exposed, and the inhibitor of the Wnt/β-catenin signaling pathway (IWR-1)^25^ was administered once a day after the exposure (Fig. 1). The periapical lesion volume was analyzed by micro-CT, showing that the periapical lesion volume in the IWR-1 group was significantly larger than that in the vehicle group at 4 weeks (vehicle group: 1.46 ± 0.01 mm^3^, IWR-1 group: 1.65 ± 0.33 mm^3^) (Fig. 2A). To confirm the suppression of the Wnt/β-catenin signaling pathway, we evaluated the expression of *Axin2* by in situ hybridization. The expression of *Axin2* was decreased in the IWR-1 group compared to that in the vehicle group after administration of IWR-1 (Fig. 2B). H–E staining results revealed the presence of heavy hematoxylin-concentrated cells around the root and the alveolar bone (Fig. 3). We observed the expression of *Runx2* and *Col1a1* on the surface of the alveolar bone around the periapical lesion in both groups, which was decreased in the IWR-1 group compared to that in the vehicle group (Fig. 3, Supplementary Fig. S1).

### Activation of the Wnt/β-catenin signaling pathway reduced the periapical lesion volume

**Figure 4:**
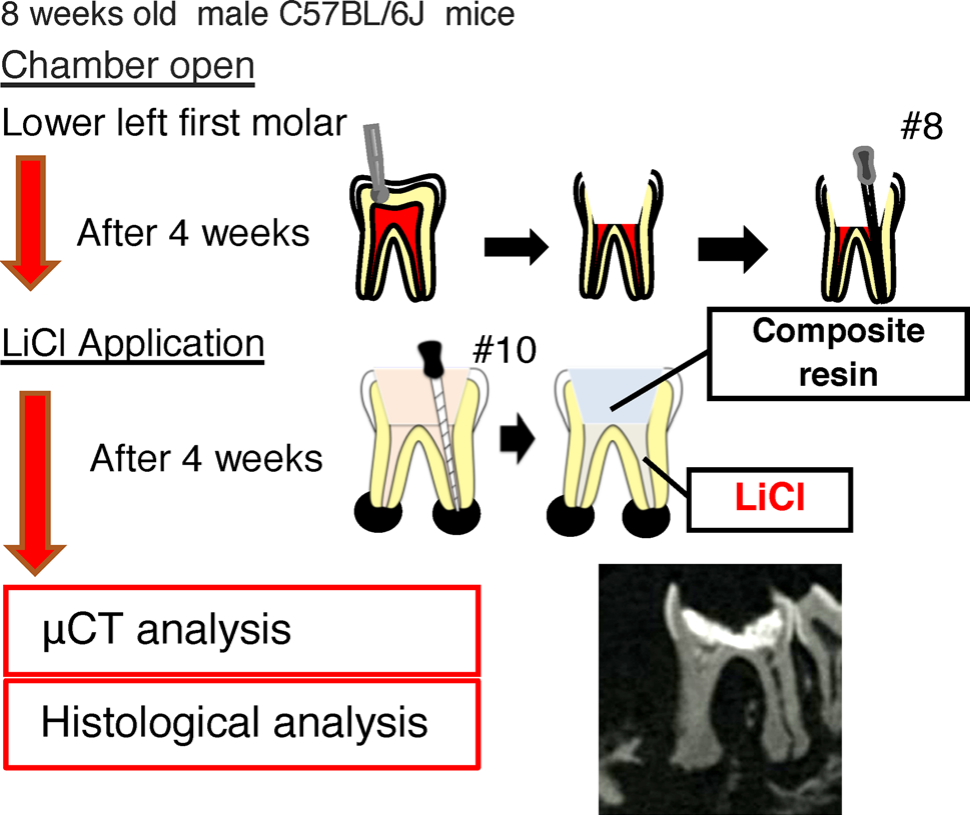
In vivo activation model of the Wnt/β-catenin signaling pathway. The pulp chamber of the lower first molar was exposed to the oral cavity. At 4 weeks after pulp exposure, root canal cleaning was performed using the # 10 K file. After root canal cleaning, LiCl was applied to the root canal, and the periapical lesions were evaluated by micro-CT and histological analysis. A representative image for the micro-CT is shown.

**Figure 5:**
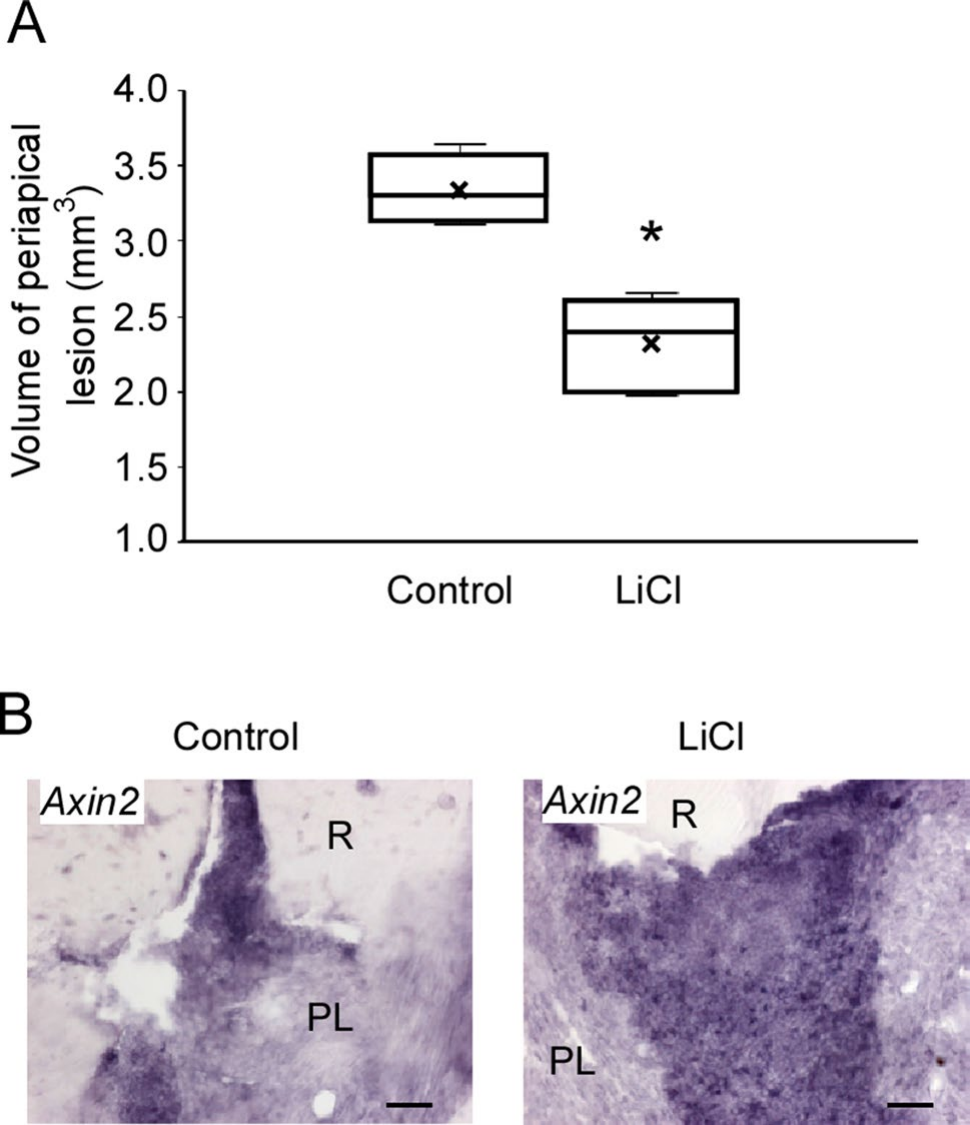
Activation of the Wnt/β-catenin signaling pathway reduced the periapical lesion volume. (**A**) Quantification of periapical lesion volumes of the control group (white bar, mean ± SD, n = 4) and the LiCl application group (black bar, mean ± SD, n = 4). Two-tailed paired Student’s *t* test, *P < 0.05. (**B**) In situ hybridization for *Axin2* in the periapical lesion at 4 weeks after chamber opening. *R* root, *PL* periapical lesion. Scale bars = 50 μm.

**Figure 6:**
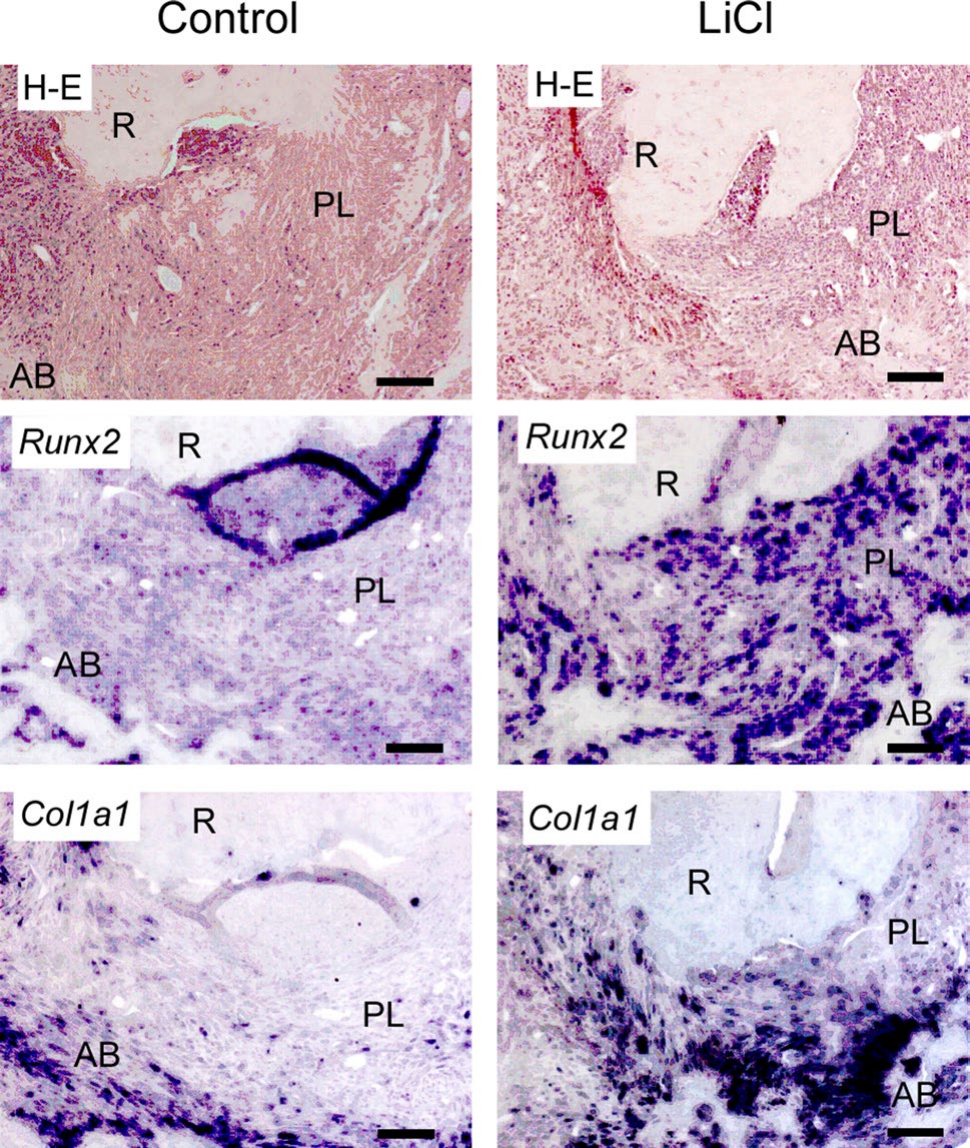
Activation of the Wnt/β-catenin signaling pathway resulted in the spreading of the expression areas of osteoblast differentiation markers. Periapical lesion tissues of the control group and the LiCl application group were subjected to H–E staining and in situ hybridization for *Runx2* and *Col1a1*. *R* root, *PL* periapical lesion, *AB* alveolar bone. Scale bars = 100 μm.

**Figure 7:**
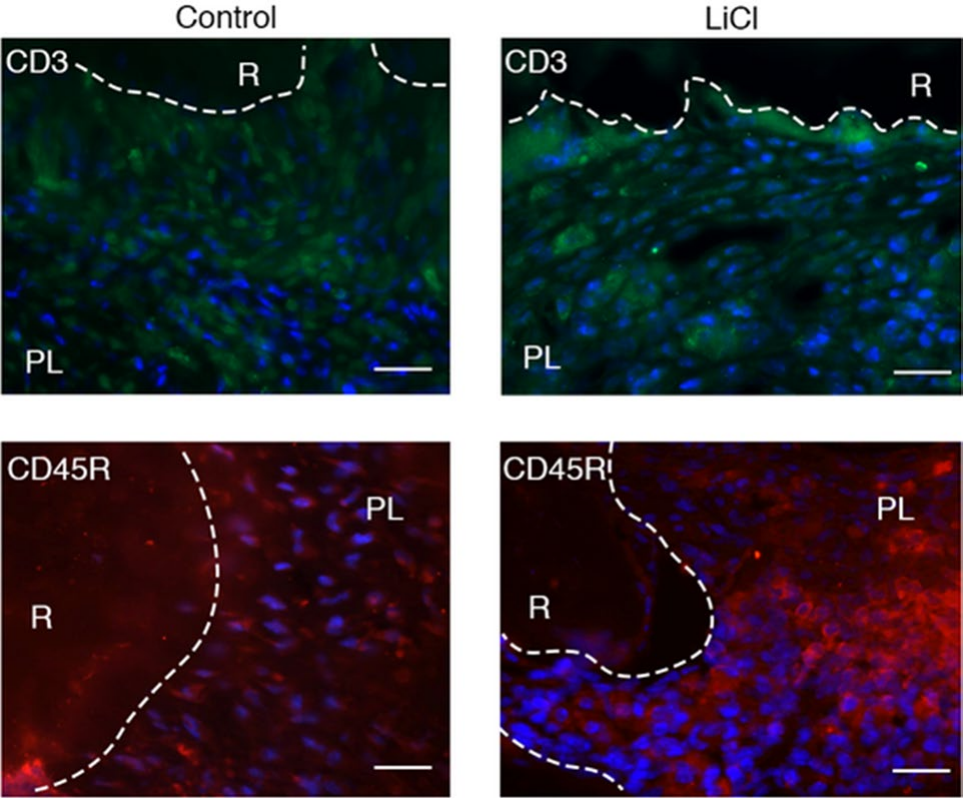
A large number of CD45R-positive cells were observed in the LiCl application group. Periapical lesion tissues of the control group and the LiCl application group were visualized by staining with Alexa 488 (CD3), Alexa 594 (CD45R), and DAPI. *R* root, *PL* periapical lesion. Scale bars = 50 μm.

LiCl is a chemical known to activate the Wnt/β-catenin signaling pathway^21^. Hence, we applied it to the root canal for 4 weeks after exposure, followed by histological analysis and micro-CT analysis (Fig. 4). The periapical lesion volume in the LiCl application group was significantly smaller than that in the control group at 4 weeks after root canal treatment (control group: 3.44 ± 0.30 mm^3^, LiCl application group: 2.32 ± 0.30 mm^3^) (Fig. 5A). To confirm the activation of the Wnt/β-catenin signaling pathway, we evaluated the expression of *Axin2* by in situ hybridization. We observed that the LiCl application group showed a higher expression level of *Axin 2* than that in the control group (Fig. 5B). The results of H–E staining of fibroblasts and angiogenesis appeared in both groups (Fig. 6). In the control group, the expression areas of *Runx2* and *Col1a1* were observed only in the bone surface surrounding the periapical lesion (Fig. 6). Conversely, in the LiCl application group, the expression areas of *Runx2 and Col1a1* were observed not only in the bone surface surrounding the periapical lesion but also in the periapical lesion (Fig. 6). There was no difference in CD3-positive T cells between the two groups (Fig. 7). In contrast, many CD45R-positive B cells were observed in the LiCl application group compared to that in the control group (Fig. 7).

## Discussion

In clinical cases, some patients have no radiolucent area in the periapical lesion despite the apparent occurrence of bacterial infection in the root canal. Conversely, there are also some patients who show a radiolucent area in the periapical lesion despite tight root canal filling. Based on these various clinical cases, we speculated that some factors other than bacterial infection are involved in the onset of periapical periodontitis. Periapical periodontitis is caused by the immune response to foreign factors, such as bacterial invasions, resulting in the subsequent absorption of the alveolar bone by osteoclasts, induced by the production of inflammatory cytokines during the immune response. Thus, periapical periodontitis is caused by bacterial infection factors and host factors such as immune responses and bone metabolism. Recently, focusing on this host fact, several disease-related genes have been identified using SNPs for various multifactorial diseases. Therefore, we decided to analyze the genes related to periapical periodontitis. We chose to extract a candidate gene related to immune response and bone metabolism, i.e., *LRP5*, a gene involved in the bone metabolism system^9–18^. We found that the distribution of *LRP5* SNP (rs3736228) was significantly biased in hetero types (Table 1). Researchers reported that *A1330V* is associated with T cell factor/lymphoid enhancer factor family (Tcf/Lef) activity in an in vitro study^18^. Therefore, in this study, we successfully identified a new SNP associated with the onset of periapical periodontitis.

To address the mechanism and factors related to periapical periodontitis, we used a recently-developed murine periapical periodontitis model^26,27^. Recently, some factors related to periapical periodontitis have been clarified^26,27^. From the result of our SNP analysis, we try to clarify the role of the Wnt/β-catenin signaling pathway for periapical periodontitis. First, we analyzed the volume of the periapical lesion when the Wnt/β-catenin signaling pathway was suppressed. IWR-1, a low-molecular-weight compound, can penetrate the cell membrane easily and effectively inhibits cellular signaling pathways^25^. IWR-1 stabilizes the complex of Axin2, APC, Ck1, and Gsk3β and then promotes the degradation of β-catenin. In the present study, an enlarged volume of the periapical lesion was observed in the IWR-1 group compared to that in the vehicle group (Fig. 2A). Therefore, we performed in situ hybridization to verify whether the enlarged periapical lesion volume in the IWR-1 group was caused by the inhibition of the Wnt/β-catenin signaling pathway by IWR-1. We found that *Axin2* expression was suppressed in the IWR-1 group (Fig. 2B). Furthermore, the expression levels of *Col1a1* and *Runx2*, osteoblast differentiation markers, were decreased in the IWR-1 group (Fig. 3, Supplementary Fig. S1). There was no change in osteoclast differentiation (Supplementary Fig. S2). These results demonstrated that the inhibition of the Wnt/β-catenin signaling pathway by the administration of IWR-1 suppressed the differentiation of osteoblasts and then promoted the enlarged volume of the periapical lesion.

The inhibition of the Wnt/β-catenin signaling pathway resulted in the enlargement of the periapical lesion. According to the IWR-1 experiment results, we speculated that the activation of the Wnt/β-catenin signaling pathway reduced the volume of the periapical lesion volume. We next explored the relationship between the activation of the Wnt/β-catenin signaling pathway and the volume of the periapical lesion. It is known that LiCl activates the Wnt canonical pathway. LiCl also inhibits the activity of GSK3β, induces the accumulation of β-catenin in the cytoplasm, and activates transcription by promoting the intranuclear translocation of β-catenin. Therefore, we examined whether LiCl exhibits a healing ability for periapical periodontitis using a murine periapical periodontitis model^26,27^. The periapical lesion volume in the LiCl application group was significantly reduced after 4 weeks of LiCl application (Fig. 5A). Next, we performed in situ hybridization to verify whether the reduced periapical lesion volume observed in the LiCl application group was caused by the activation of the Wnt/β-catenin signaling pathway by LiCl. Our results showed that the tissue sections at 24 h after LiCl application demonstrated high expression of *Axin2* (Fig. 5B). This result suggested that LiCl activates the Wnt/β-catenin signaling pathway in the periapical lesion. In addition, LiCl application resulted in a higher expression of *Col1a1* and *Runx2* than that in the control group (Fig. 6, Supplementary Fig. S1). These results demonstrated that the activation of the Wnt/β-catenin signaling pathway by LiCl induces the differentiation of osteoblasts and promotes the healing of periapical periodontitis.

Because the Wnt canonical pathway regulates the differentiation of immune cells, we analyzed the effect of LiCl application on the differentiation of immune cells. Although no difference in the number of CD3-positive cells between the LiCl application group and the control group (Fig. 7) existed, the number of CD45R-positive cells in the LiCl application group was higher (Fig. 7). CD45R is expressed from pro-B cells to mature B cells, and a previous study showed that the Wnt/β-catenin signaling pathway positively regulates the proliferation ability of B cells^23^. This is consistent with the result of the increase in the number of B cells in the periapical lesion of the LiCl application group. Although LiCl reportedly maintains T cells in an undifferentiated state^24^, no change occurred in CD3-positive cells. This discrepancy might be caused due to the different application methods, i.e., the local application into the root canal in this study and the systemic administration in the previous study^24^.

In summary, LiCl promotes the healing of periapical periodontitis by inducing bone formation and immune responses. Although conventional root canal treatment agents such as calcium hydroxide primarily target the disinfection of bacteria in the root canal, LiCl targets the Wnt/β-catenin signaling pathway by regulating bone metabolism and immune response. Therefore, our results suggest that LiCl could be used as a bioactive next-generation root canal treatment agent.

## Methods

### Sample population and genotyping

This study was approved by the research Ethics Review Committee of Osaka University (#450), Osaka, Japan and the Ethics Review Committee of Osaka University Dental Hospital (#H25-E22). All participants signed an informed consent and provided a sample as a source of genomic DNA. Participants whose radiographic records showed periapical lesions measuring > 3 mm in diameter were assigned to the case group (n = 50). Participants who presented with no periapical lesions after root canal treatment were assigned to the control group (n = 30). Buccal mucosa was collected by wiping with a sterile swab. The tip of the swab was immersed in a lysis buffer [100 mM Tris–HCl (pH 8.5), 5 mM EDTA, 0.2% SDS, 200 mM NaCl, 100 μg proteinase K/ml] and reacted at 55 °C for 24 h. After the reaction, genomic DNA was prepared by isopropanol precipitation and ethanol precipitation. Genomic DNA was diluted in 100 μl TE solution and stored at − 80  °C. PCR was performed for *LRP5* (dbSNP ID: rs3736228) gene amplification using TaqMan GTXpressTM Master Mix and TaqMan SNP Genotyping Assays (Applied Biosystems, California, USA). All methods were performed following the relevant guidelines and regulations set forth by the Declaration of Helsinki. Informed written consent was obtained from all participants.

### Murine periapical periodontitis model

The study was approved by the research Ethics Committee of the Osaka University, Osaka, Japan, and all experiments were performed according to the guidelines related to animal care (AD-26-011-0) and compliant with ARRIVE guidelines (http://www.nc3rs.org.uk/page.asp?id=1357). C57BL/6 J mice (8-week-old) were intraperitoneally anesthetized with Domitor (0.3 mg/kg) (Nippon Zenyaku Kogyo Co., Fukushima, Japan), Dolmicam (4 mg/kg) (Astellas Pharma Inc., Tokyo, Japan), and Betferar (5 mg/kg) (Meiji Seika Pharma, Tokyo, Japan). The pulp chamber of the lower first molar was accessed with a # 1/4 round bar (Dentsply, Ballaigues, Switzerland) equipped with an electric engine (VIVA MATE G 5, NSK, Tochigi, Japan). The root canals were instrumented with the # 08K file (Dentsply) under the operating microscope and left exposed to the oral cavity. Mice were monitored daily for clinical signs of abnormal posture, lack of grooming, weight loss exceeding 20% of body weight, and anorexia. Though the presence of any of these findings was considered as an endpoint and mice would be euthanized using a CO_2_ chamber, we did not observe any of these findings in the mice used for experiments.

### IWR-1 administration

The solutions (0.04 ml) adjusted as follows were administered into the tail vein once a day from the day of pulp exposure (Fig. 1). The solutions were adjusted as follows: control solution: DMSO (Wako Pure Chemical Industries, Osaka, Japan) was diluted in phosphate-buffered saline (PBS) (Final concentration: 5%). IWR-1 (Sigma-Aldrich, Missouri, USA) solution (IWR-1: 2.5 μmol/kg) was diluted in the control solution. At 4 weeks after the first administration, the periapical lesion volume was measured (each group, n = 4).

### LiCl application

At 4 weeks after pulp exposure, root canal cleaning was performed using a # 10 K file (Dentsply). LiCl was ground into the small size powder within 100 μm. After root canal cleaning, LiCl (Wako Pure Chemical Industries, Ltd.) was applied into the root canal (0.025 g per each root canal as a powder) with the slightly moistened # 10 K file (Dentsply) under the operating microscope. Root canals of the control group did not contain anything. The pulp chamber was closed with the bonding material Clear fill bond SE ONE^®^ (Kuraray Noritake Dental, Tokyo, Japan), and a composite resin (MI flow: GC, Tokyo, Japan).

### Micro-CT measurement

The volume of the periapical lesion was measured by micro-computed tomography (CT) (R_mCT 2: Science Mechatronics, Tokyo, Japan) performed on the lower first molar. The photographing conditions were set as follows: tube voltage 90 kV, tube current 160 μA, and slice width 5 μm. The obtained images were analyzed using SimpleViewer software (Science Mechatronics). Based on the method described by Kalatizis-Sousa et al.^28^ and, Yoneda et al.^29^, the periapical lesion volume was calculated using the bone morphometry software (TRI 3D-BON: RATOC, Osaka, Japan). The lesion volume was defined as the periapical transmission image volume and compared between experimental groups as previously reported^27^. The Student’s *t* test evaluated the statistically significant differences in the lesion volume (α = 0.05).

### Sample preparation for histological analysis

After subjecting the mice to the experiments described above, they were reflux-fixed in 4% paraformaldehyde (PFA) solution. The mandibles were collected, immersed, and fixed in 4% PFA solution for 24 h, followed by demineralization with 10% EDTA solution for 2 weeks. After demineralization, the samples were dehydrated with an ascending ethanol series and embedded in paraffin. Then, thin slices with a thickness of 9 μm were prepared.

### Hematoxylin–eosin staining

Paraffin sections were deparaffinized, washed with water, and reacted with Mayer’s hematoxylin solution (Muto, Osaka, Japan) for 7 min. Then, the sections were washed with running water for 20 min and stained with eosin solution (Merck, Darmstadt, Germany) for 5 min, dehydrated with ethanol and decolorized, penetrated with xylene, and sealed with 50% glycerol/PBS. The apical lesion and the surrounding bone were observed under an optical microscope (Axioskop 2 plus; Carl Zeiss, Aalen, Germany).

### In situ hybridization

Paraffin sections were deparaffinized, washed with 0.01 M PBS, fixed with 4% PFA for 10 min, and washed again with PBS. The sections were reacted with 1 μg/ml protease K (Takara Bio, Shiga, Japan) for 5 min and then post-fixed with 4% PFA. This was followed by acetylation with 0.1 M triethanolamine containing 0.25% acetic anhydride and washing with 0.01 M PBS. Prehybridization was carried out at 55 °C for 1 h, and hybridization was carried out overnight at 70 °C using digoxigenin-labeled RNA probes, *Axin2*; addgene #21277, NM_015732, nt1774-2787, *Col1a1*; M_007742, nt29553415, *Runx2*; AF010284, nt922_1746. After hybridization, the sections were placed in fivefold concentration sodium citrate solution (5-SSC) for 20 min. They were reacted with 0.2-SSC at 70 °C for 20 min and left to stand for 5 min in 0.2-SSC in maleate buffer (MBA) for 5 min. Then, blocking was performed for 2 h with a blocking solution containing 5% goat serum (Vector Laboratories, California, USA). After reacting with alkaline phosphatase (AP)-labeled anti-digoxigenin antibody (1:5000) (Roche, Basel, Switzerland) at 4 °C for 24 h, the sections were washed with MBA supplemented with 0.1% Tween 20, followed by washing with distilled water. The sections were reacted with BM Purple AP (Roche) as a substrate for 6 h at room temperature, after which they were washed with PBS and sealed with 50% glycerol/PBS. The apical lesion and the surrounding bone were observed under an optical microscope.

### Immunohistochemistry

The paraffin sections were deparaffinized, washed with Tris-buffered saline (TBS), reacted at 100 °C for 10 min in a citrate buffer, and allowed to stand at room temperature for 30 min. After blocking for 1 h with 10% goat serum-containing blocking solution (10% goat serum/TBS), each antibody was reacted overnight at room temperature. The concentration of each antibody was 1:100 for CD3 (ab16669: Abcam, Cambridge, United Kingdom) and 1:50 for CD45R (ab64100: Abcam) for the experiments. After reaction with the primary antibody, the sections were washed with TBS and incubated with 1:500 Alexa 488-goat anti-rabbit IgG (Invitrogen, California, USA) or 1:500 Alexa 594-goat anti-rat IgG (Abcam) for 2 h. Then, the sections were washed and reacted with DAPI (4′,6-diamidino-2-phenylindole) (Sigma) for 15 min. After the reaction, they were washed and sealed with 50% glycerol. The apical lesion and the surrounding bone were visible under a fluorescence microscope.

### Statistical analyses

Results are presented as mean ± SD. For SNP analyses, comparisons between groups were performed using Fisher’s exact test (α = 0.05). The Student’s *t*-test quantified the differences in the periapical lesion volume.

## Supplementary Information

Supplementary Legends.

Supplementary Figure S1.

Supplementary Figure S2.
