# Supplementary material for: The Wnt/β-catenin signaling pathway has a healing ability for periapical periodontitis
Source: Sci Rep. 2021 Oct 4;11:19673. doi: 10.1038/s41598-021-99231-x (PMC8490427; doi:10.1038/s41598-021-99231-x)
Supplement: Supplementary file 1 — Supplementary Legends. [file 41598_2021_99231_MOESM1_ESM.docx]

**Supplementary information figure legends**

**Figure S1.** Quantification of *in situ* hybridization (ISH) signals of (A) Figure 3 and (B) Figure 6. Quantification of ISH signal intensity was performed using BoneJ, which is a plugin software for ImageJ (Doube M, Kłosowski MM, Arganda-Carreras I, Cordeliéres F, Dougherty RP, Jackson J, Schmid B, Hutchinson JR, Shefelbine SJ. (2010) BoneJ: free and extensible bone image analysis in ImageJ. *Bone* 47:1076-9. [doi: 10.1016/j.bone.2010.08.023](http://dx.doi.org/10.1016/j.bone.2010.08.023)). Firstly, the images were captured with a similar gain condition. Then, each image was assembled as a single-layered image. Three to four regions at the root apical domain were randomly selected, and binarized images were generated in ImageJ software. Signal area/total area was automatically measured by the fraction tool in BoneJ software. Student’s *t*-test, *: *P* < 0.05.

**Figure S2.** There was no change in osteoclast differentiation between the vehicle group and the IWR-1 treatment group. Paraffin sections were deparaffinized, washed with water, and reacted with tartrate-resistant acid phosphatase (TRAP). R: Root, PL: Periapical lesion, AB: Alveolar bone. Scale bars = 100 μm.
