# Supplementary figures and images for: The Wnt/β-catenin signaling pathway has a healing ability for periapical periodontitis
Source: Sci Rep. 2021 Oct 4;11:19673. doi: 10.1038/s41598-021-99231-x (PMC8490427; doi:10.1038/s41598-021-99231-x)

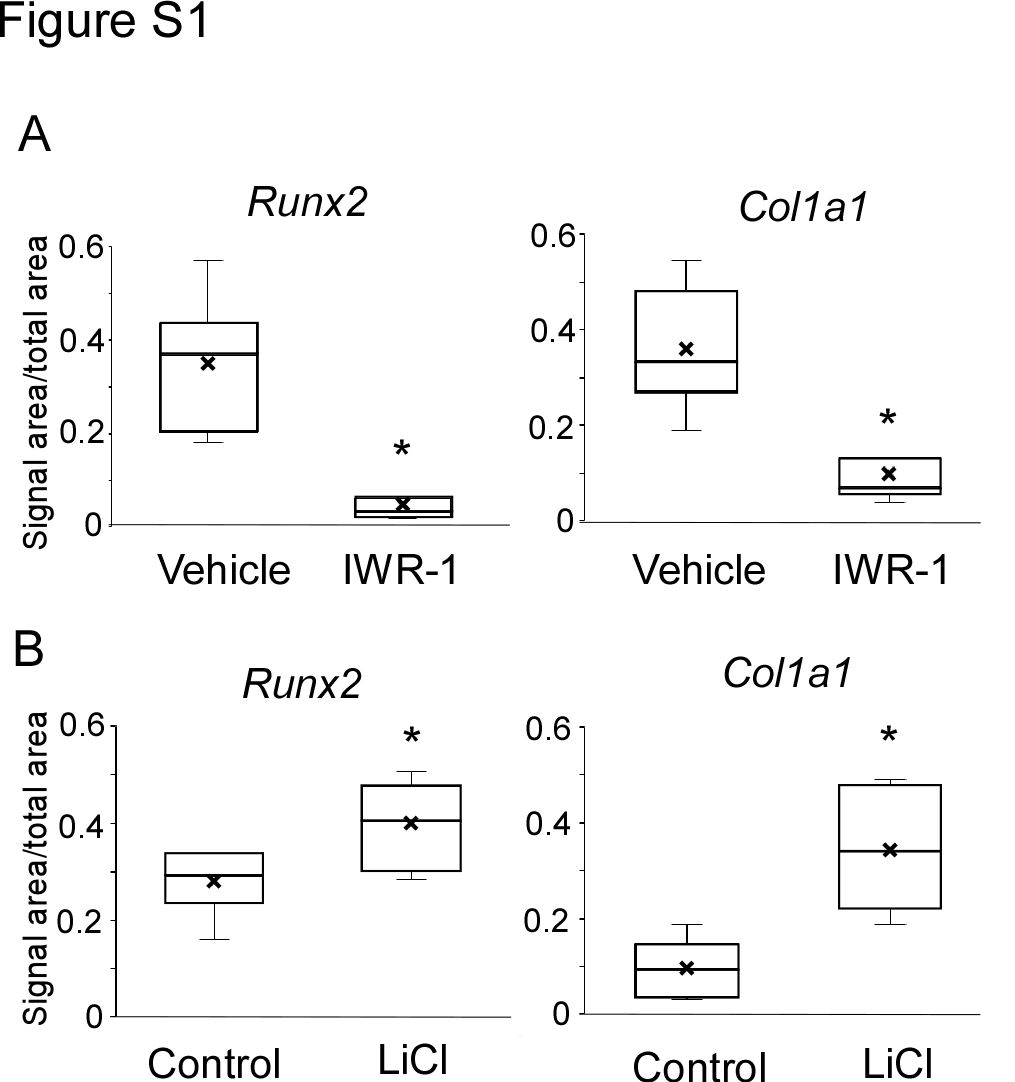

Supplement: Supplementary file 2 — Supplementary Figure S1. [file 41598_2021_99231_MOESM2_ESM.tif]

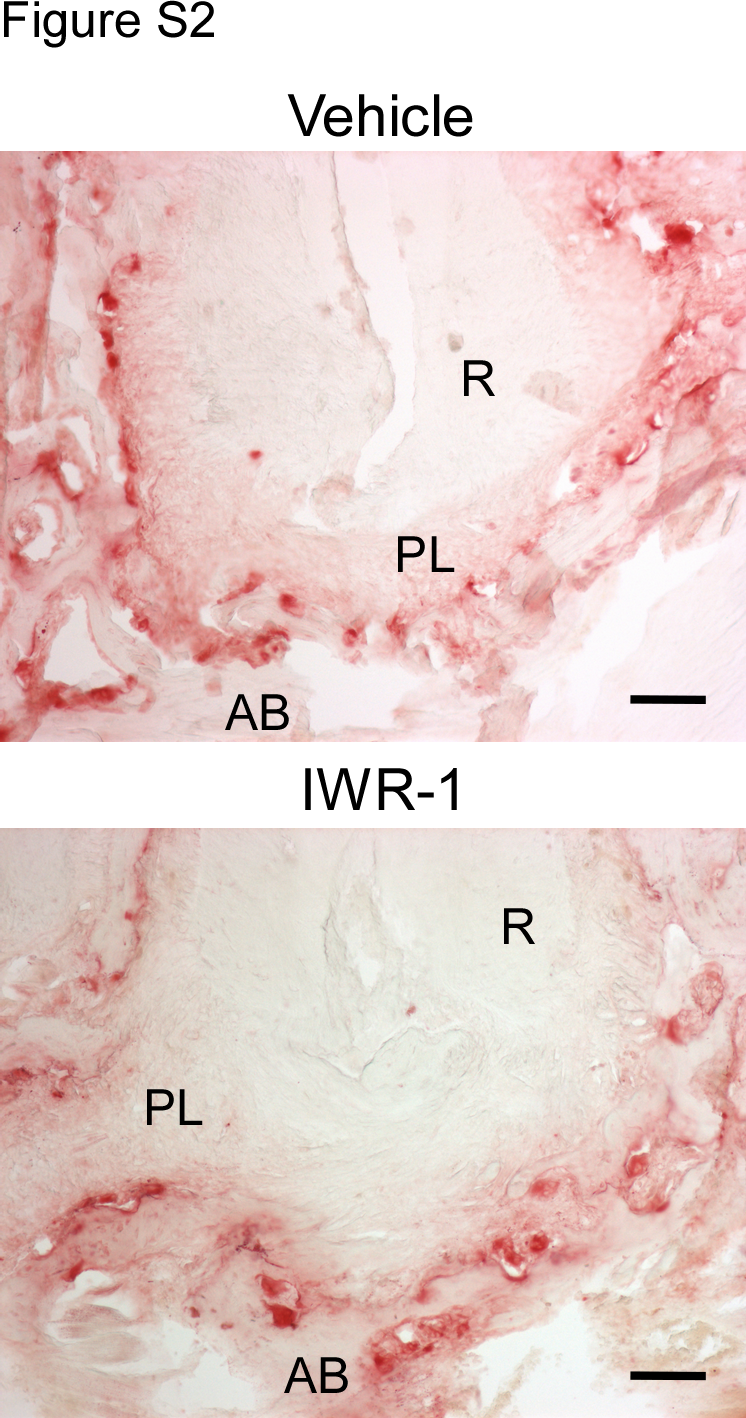

Supplement: Supplementary file 3 — Supplementary Figure S2. [file 41598_2021_99231_MOESM3_ESM.tif]
